# Tunable High‐Performance Metamaterial Filters Based on Novel SRR Architectures

**DOI:** 10.1002/advs.75552

**Published:** 2026-05-07

**Authors:** Lingxi Qu, Liya Zheng, Ruopeng Liu, Chunlin Ji, Bin Li

**Affiliations:** ^1^ School of Materials Shenzhen Campus of Sun Yat‐Sen University Shenzhen China; ^2^ Kuang‐chi Institute of Advanced Technology Shenzhen China

**Keywords:** 3D printing, bandpass filters, electromagnetic filtering mechanisms, metamaterials

## Abstract

Metamaterials, known for their unique electromagnetic properties and lightweight design, are ideal for applications requiring high electromagnetic interference resistance and enhanced maneuverability. This study designed and fabricated four novel tunable passband metamaterial filters with ultra‐thin ceramic substrates, combining both simulation and experimental approaches. The SM‐2 model achieves over 97% electromagnetic wave transmittance, with surface reflectance below 0.017% at its resonant frequency, while maintaining a relative thickness of just 0.06 *λ*_L_. Furthermore, by adjusting the unit cell geometry, SM‐1 demonstrates a passband coverage of nearly 70%. Simulations identify two distinct filtering mechanisms: one driven by capacitive gain from local field superposition and the other by capacitive‐inductive impedance matching, both of which enable efficient, low‐loss transmission of electromagnetic energy. These metamaterials hold great potential for precise signal transmission and enhanced mobility in lightweight systems, making them promising candidates for advanced electromagnetic filtering systems.

## Graphical abstract

Based on two novel SRR configurations and two independent SRR integration approaches, four tunable bandpass metamaterial filters were fabricated with an ultra‐thin ceramic substrate. Among them, SM‐2 achieves over 97% electromagnetic wave transmittance, with a minimal 1.15% deviation in its resonant frequency prediction, making them promising candidates for advanced electromagnetic filtering systems.

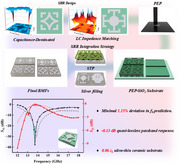

## 1 Introduction

Recent advancements in hypersonic and high‐maneuverability vehicles highlight the growing need for materials that can withstand electromagnetic interference and adapt to dynamic flight paths [1, 2]. Consequently, materials combining electromagnetic filtering properties with lightweight designs are essential. Metamaterials, as engineered composites with periodic subwavelength structures, have garnered attention for their unique electromagnetic properties, offering capabilities not found in conventional materials [3, 4, 5]. These materials enable precise control over electromagnetic wave propagation, making them indispensable in a wide range of applications, including convertors, suppressors, sensors, and meta‐lenses [6, 7, 8, 9, 10, 11, 12, 13, 14]. Furthermore, their ability to miniaturize devices has led to applications in fields such as solar energy harvesting, biomedical imaging, radar stealth technology, and electromagnetic shielding [15, 16, 17, 18, 19, 20, 21]. The integration of metamaterial‐based filtering components in next‐generation aerospace and communication systems has already demonstrated improvements in signal integrity, energy efficiency, and ensured stable electromagnetic performance in complex and challenging environments [2, 22, 23].

However, traditional metamaterial fabrication methods, such as photolithography and etching, are limited to two‐dimensional structures and face challenges in producing three‐dimensional configurations. For instance, Zhao et al. developed a broadband metamaterial filter (BMF) based on printed circuit board (PCB) technology, achieving a thickness of about 0.05 *λ*_L_ while maintaining both a wide passband and angular stability [24]. Despite the advancements in high‐precision metamaterial fabrication, the use of polymer substrates, which are commonly employed due to material compatibility, restricts their application in high‐temperature environments. Additive manufacturing (AM) is a promising alternative, offering greater design flexibility and the ability to create complex, intricate structures [2, 25, 26, 27, 28]. AM allows metamaterial substrates to move beyond polymer‐based materials by utilizing ceramics with higher thermal stability and mechanical strength. This opens up new applications in high‐temperature environments. For instance, Dumene et al. achieved the integration of complex alumina metamaterial structures through light‐curing 3D printing, improving the dielectric loss of the metamaterials by controlling the printing parameters [29].

Since the theoretical prediction of “left‐handed materials” by Veselago in 1968 [30] and Pendry's 2006 design methodology for metamaterials [31], the understanding of metamaterial electromagnetic behavior has advanced significantly. The design of the bandpass metamaterial filter unit cell (BMF UC) has been guided by theoretical models, including interference theory, impedance matching, transmission line theory, and coupled mode theory, which have been empirically validated [32, 33, 34, 35]. The split‐ring resonator (SRR) structure, a foundational element in many BMF designs, has evolved to meet various application needs, often relying on the magnetoelectric coupling resonance [36, 37]. Beyond microwave and Ku‐band implementations, resonator‐based metamaterial structures have also been extensively studied in the terahertz (THz) regime, where subwavelength resonators exhibit tunable spectral responses, strong field confinement, and high‐sensitivity filtering behavior [38, 39, 40, 41]. These studies further validate the resonance‐driven electromagnetic mechanisms underlying SRR‐type structures and highlight the influence of fabrication‐dependent factors on spectral performance. At frequencies closer to the present work, recent studies have demonstrated Ku‐ and K‐band absorbers and multi‐band double‐negative metamaterial configurations employing symmetrical circular SRR variants and hybrid resonator combinations, further illustrating the structural versatility and frequency scalability of SRR‐based metamaterial designs [42, 43]. Despite these structural variations across frequency regimes, the fundamental filtering response of SRR‐based systems remains governed by resonance‐driven charge redistribution and inductor‐capacitor (*LC*) interactions. When exposed to an alternating electric field (*E‐*field), the SRR experiences periodic charging and discharging, causing phase shifts between voltage and current. At the resonant frequency (*f*_0_), inductive and capacitive impedances of the SRR's *LC* circuit achieve equilibrium, maximizing electromagnetic transmission efficiency [44, 45]. In compact design applications, the coupled spiral resonator (CSR) not only ensures a constrictive passband window but also allows for precise control of the *f*_0_ by adjusting the spiral length, enhancing design flexibility. [Correction statement added on 11 May 2026 after first online publication: typographical error of “LC” is updated in this version.]

**FIGURE 1:**
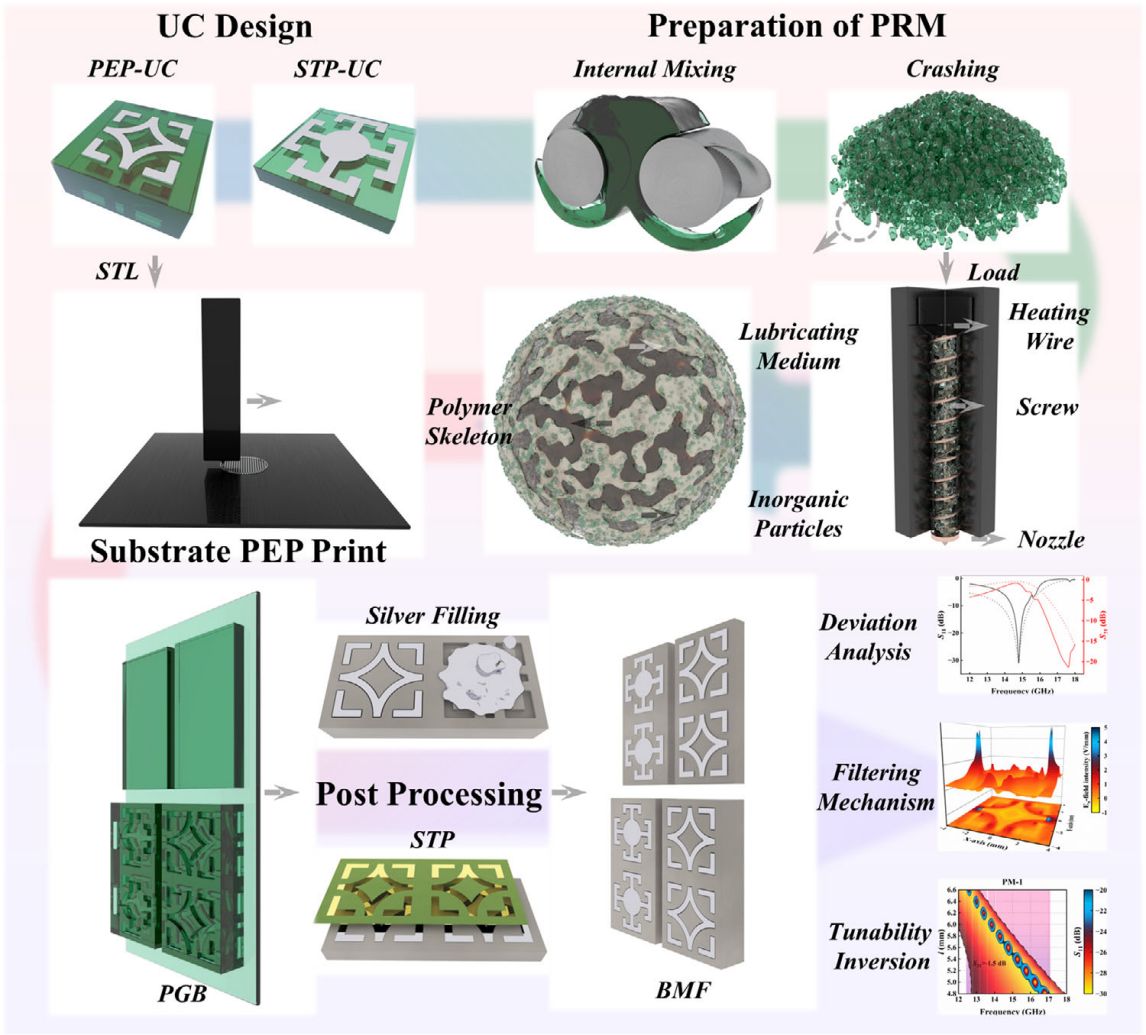
Simulation guiding experimental design concept.

The simulation guiding the experimental design concept of this work is shown in Figure 1. Most reported Ku‐band SRR‐based metamaterial bandpass filters rely on split‐gap *LC*‐resonance, where passband tuning is mainly achieved through geometric scaling or gap/coupling adjustments. In contrast, this study proposes two structurally distinct resonators, including the coupled closed‐loop resonator (CLR) and the CSR, which implement two distinct transmission mechanisms within a single Ku‐band platform. The CLR emphasizes capacitance‐dominated transmission gain via local field superposition, while the CSR utilizes a centrosymmetric spiral to achieve *LC* impedance matching. Based on the two SRR preparation processes of silver filling and screen transfer printing (STP), four different BMF‐UC models were designed and fabricated to evaluate fabrication‐induced performance variations. SiO_2_, with its excellent dielectric properties and thermal stability, is well‐suited for meeting the low‐loss transmission requirements of high‐frequency signals [46, 47]. To enhance the out‐of‐band suppression in the BMF design for this work, a small amount of fused glass was incorporated. This addition increases the dielectric loss within the stopband through mechanisms like conductance loss, while also reducing the sintering temperature of the substrate. Three‐dimensional ceramic substrates were prepared via powder extrusion printing (PEP), enabling integration of the SRR architectures into thermally stable SiO_2_‐based ceramics. The two SRR integration routes impose different substrate thickness constraints. Groove‐based silver filling requires thicker substrates for structural integrity, whereas groove‐free STP enables ultra‐thin substrates down to 0.06 *λ*_L_, while also improving SRR pattern fidelity and fabrication consistency. Reducing the substrate thickness decreases the interlayer spacing between adjacent SRR layers, thereby altering the electromagnetic coupling strength and resonance behavior of the structure. Such coupling regulation directly translates into enhanced filtering performance, as experimentally evidenced by electromagnetic wave transmittance exceeding 97% at *f*_0_ and passband tunability spanning up to 70% of Ku‐band. The combination of mechanism‐differentiated SRR designs, ultra‐thin ceramic additive manufacturing, and complementary integration strategies establishes a lightweight, thermally stable Ku‐band metamaterial filtering platform. Beyond electromagnetic performance, the ultra‐thin substrate design further reduces overall system weight, offering additional advantages for maneuvering‐ or orbit‐adjustment‐sensitive communication systems operating in extreme environments.

## 2 Results and Discussion

### 2.1 BMF Design

**FIGURE 2:**
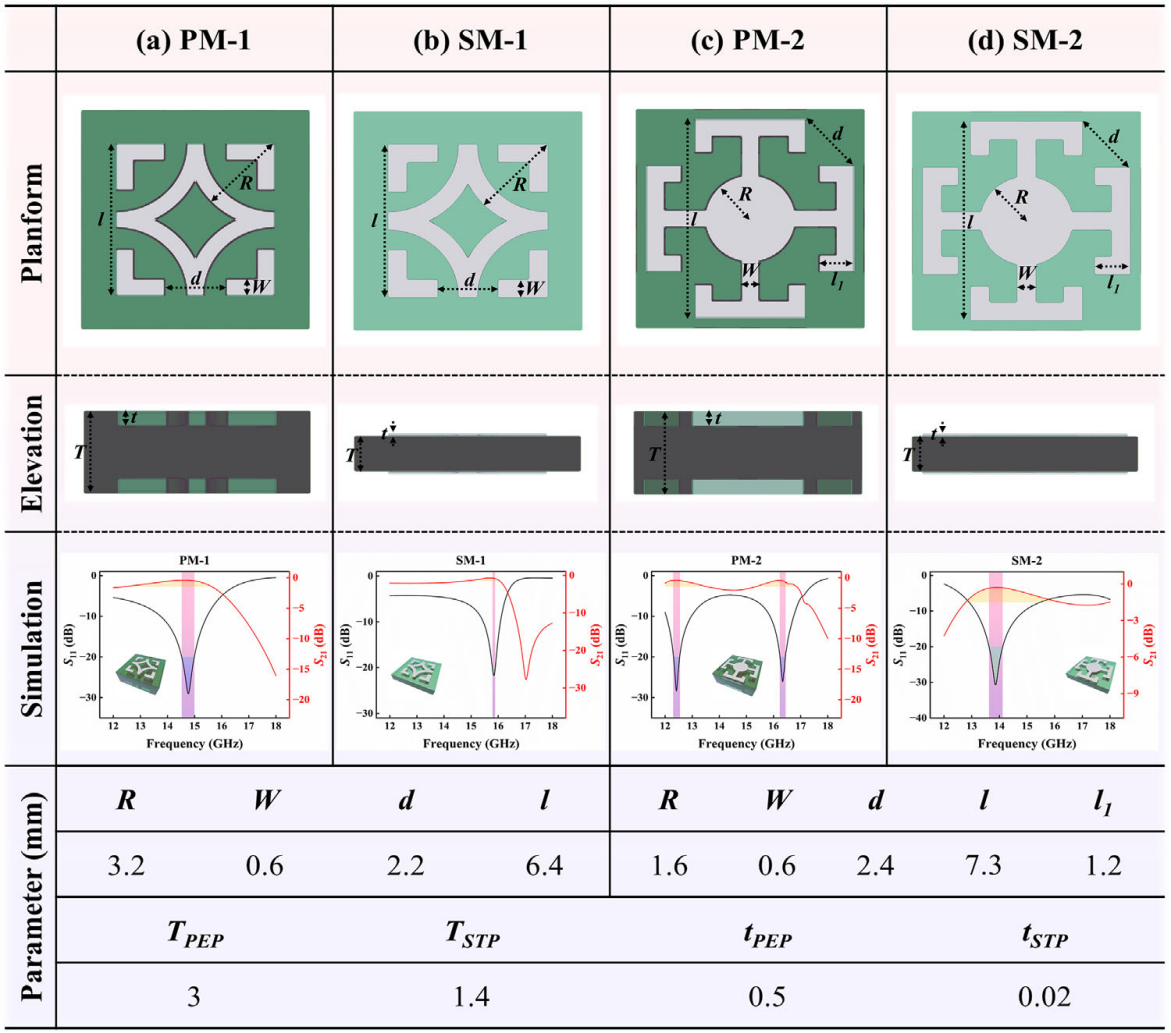
Structural configurations, geometric parameters, and simulated filtering responses of four BMF unit‐cell models: (a) PM‐1, (b) SM‐1, (c) PM‐2, and (d) SM‐2. The first and second rows show the parametric planform and elevation configurations with labeled geometric parameters and layer thicknesses. The third row presents the simulated *S*‐parameter responses (*S*_11_ and *S*_21_) as a function of frequency within the Ku‐band. The bottom table summarizes the structural parameters and fabrication‐related thicknesses (*R*, *W*, *d*, *l*, *l_1_*, *T_PEP_*, *T_STP_*, *t* *_PEP_*, and *t_STP_*).

The geometric design of the four UCs is shown in Figure 2. The characteristic frequency of the CLR in PM‐1 is mainly controlled by the coil length *l*. The filtering mechanism of CLR is significantly different from that of CSR based on the inductance and capacitance coupling effect. According to Maxwell's equations, the equivalent inductance *L* of an *LC* circuit is influenced by the coil length *l*. In the design for PM‐1 and PM‐2, the effective radius *r* of the SRR coil can be approximated as *n* times that of *l*, and the corresponding *L* is given by:

(1) $$L=\frac{\mu_{0}\pi r^{2}}{l}=\frac{\mu_{0}\pi }{l}\;\cdot {\left(n\cdot l\right)}^{2}\propto K_{1}\cdot l$$

where *µ*_0_ is the vacuum permeability. According to Gauss’ law, the *C* is determined by the aperture width *d* and the effective plate area *S*. Similarly, the coupled metal layers and the CSR's aperture can be modeled as a parallel plate capacitor, and the *S* can be expressed as *S* = *W* · *t*. The *C* is therefore:

(2) $$C=\frac{\varepsilon_{r}S}{4\pi \mathrm{kd}}=\frac{\varepsilon_{r}}{4\pi \mathrm{kd}}\;\cdot W\cdot t\propto K_{2}\cdot \frac{W\cdot t}{d}$$

where *ε*_r_ is the relative dielectric constant of the medium, and k is the electrostatic constant. Given the lack of apertures in CLR to provide capacitance, the tuning ability of *d* to its *f*_0_ is limited. Finally, based on the *LC* circuit of the conventional SRR, the relationship between the *f*_0_ and *L* and *C* is given by:

(3) $$f_{0}=\frac{1}{2\pi \sqrt{\mathrm{LC}}}\;\propto K_{3}\cdot \frac{d}{l\cdot W\cdot t}$$

These relationships confirm that tuning the SRR geometry directly modulates the *f*_0_ of the BMF. Based on the silver‐filling depth, substrates for PM‐1 and PM‐2 were designed with a 0.5 mm SRR thickness, resulting in an overall UC thickness of 3 mm. To reduce UC thickness further, SM‐1 and SM‐2 were designed with a 0.02 mm thick STP layer. The two STP‐UCs retain identical CLR and CSR geometries, with an overall UC thickness of 1.42 mm. Additional design parameters include the quarter outer ring radius *R* of CLR, the central disc radius *R* in CSR, the UC thickness *T*, and the width *W* and thickness *t* of SRRs.

The optimized parameter settings are depicted in Figure 2. The passband of PM‐1 spans from 14.53 to 14.99 GHz (*S*_11_ = ‐29.03 dB, *S*_21_ = −0.44 dB @ *f*_0_ = 14.77 GHz) as shown in Figure 2a, and the passband for SM‐1 ranges from 15.79 to 15.89 GHz (*S*_11_ = −21.74 dB, *S*_21_ = −0.78 dB @ *f*_0_ = 15.85 GHz) as shown in Figure 2b. Both structures show weak out‐of‐band suppression in the low‐frequency region of Ku‐band and high insertion loss (IL) in the high‐frequency region. Additionally, there is an approximate 0.97 GHz offset in *f*_0_, due to changes in *T* and *t*, with the thinner CLR in SM‐1 providing a weakened voltage gain, ultimately manifested as a reduced intensity of the *S*_11_ resonant peak and a significantly increased out‐of‐band suppression. Figure 2c shows PM‐2, which, due to its CSR centrosymmetric structure, has four compact spiral inductor coils and thicker SRRs, resulting in a dual‐passband structure within Ku‐band. The passband for PM‐2 ranges from 12.31 to 12.53 GHz (*S*_11_ = −28.37 dB, *S*_21_ = −0.42 dB @ *f*_0_ = 12.42 GHz) for the first passband, and from 16.23 to 16.43 GHz (*S*_11_ = −25.96 dB, *S*_21_ = −0.49 dB @ *f*_0_ = 16.34 GHz) for the second passband. In contrast, Figure 2d shows SM‐2, where the dual‐passband structure is more separated, resulting in improved low‐frequency out‐of‐band suppression but poorer high‐frequency suppression due to the shift toward the second passband outside Ku‐band. The passband of SM‐2 spans from 13.63 to 14.12 GHz (*S*_11_ = −30.74 dB, *S*_21_ = −0.29 dB @ *f*_0_ = 13.87 GHz).

### 2.2 Experimental Verification

**FIGURE 3:**
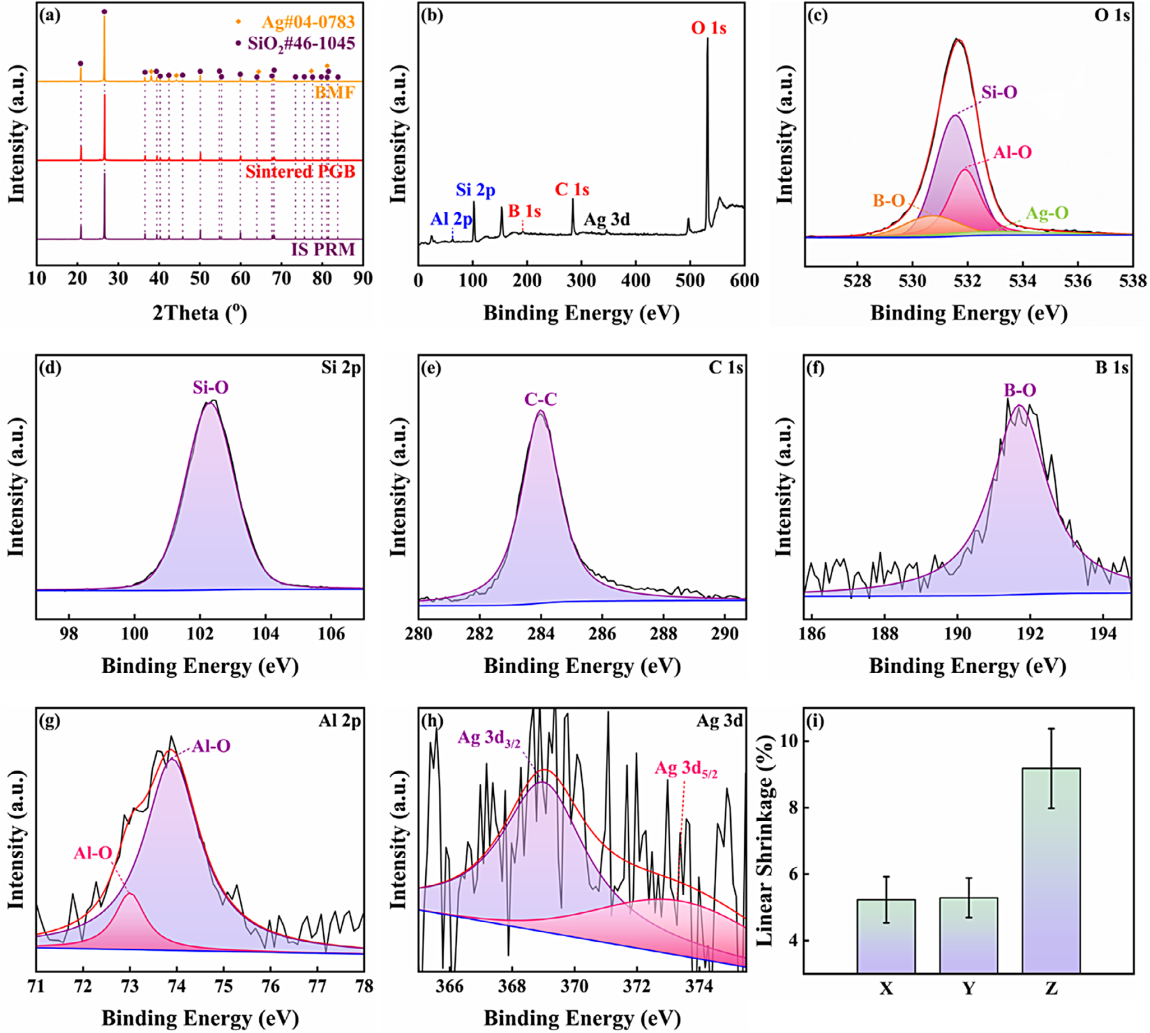
(a) XRD characterization of IS PRM, sintered PGB, and BMF samples; (b) XPS total binding energy spectrum; (c–h) Binding energy spectrum of O, Si, C, B, Al, and Ag; (i) The average linear shrinkage rate of ten PGBs.

Figure 3a shows the X‐ray diffraction (XRD) patterns of inorganic substrate printing raw material (IS PRM), the sintered printed green body (PGB), and the final BMF. All three diffraction patterns feature peaks corresponding to SiO_2_. The fused glass, which was used as a sintering aid, forms an amorphous phase, so no diffraction peaks related to it were observed. Notably, only diffraction peaks of Ag can be observed in the final BMF. Figure 3b displays the X‐ray photoelectron spectroscopy (XPS) total energy spectrum for the BMF, while Figure 3c–h displays the spectra for individual elements after peak deconvolution. The principal O peak in Figure 3c can be deconvoluted into four binding energy peaks at 530.7, 531.5, 531.9, and 533 eV, corresponding to B─O, Si─O, Al─O, and Ag─O, respectively [48]. The Si 2p spectrum (Figure 3d) shows a peak at 102.2 eV, consistent with the Si─O bond in SiO_2_, a component in the BMF substrate [49]. The C 1s spectrum (Figure 3e) reveals a C─C peak at 284.3 eV, likely from atmospheric exposure or residual carbon from polymer decomposition [50]. The B 1s spectrum (Figure 3f) indicates a B─O bond at 192 eV, while the Al 2p spectrum (Figure 3g) displays peaks at 73.0 and 73.8 eV, corresponding to Al─O bonds from trace additives in the fused glass [51, 52]. The Ag 3d spectrum (Figure 3h) exhibits characteristic peaks for elemental Ag, with spin‐orbit splitting observed at 368.2 eV (Ag 3d_3/2_) and 373.8 eV (Ag 3d_5/2_) [53, 54]. Given that the chemical shift between Ag and Ag─O is lower than the resolution of XPS [55], which leads the Ag 3d orbital signal to mask the Ag─O [56]. Only trace amounts of oxidation may be present on the silver surface, which can be detected by XPS due to its high surface sensitivity, while XRD cannot detect it. Figure 3i shows the linear shrinkage rates of ten BMF PGBs during post‐processing. To matching the dimensional shrinkage during the sintering process, PGBs closely matched the target design of a 105% magnification, with the average dimensions of 8.41 mm × 16.80 mm × 3.16 mm. After the full post‐processing sequence, the dimensions decreased to 7.97 mm × 15.91 mm × 2.87 mm, with linear shrinkage rates of 5.23%, 5.29%, and 9.18% along the *X*, *Y*, and *Z* axes, respectively. The significant shrinkage along the *Z* axis is mainly due to the loss of the polymer skeleton after pyrolysis, which leaves the inorganic particles to compact under gravity.

**FIGURE 4:**
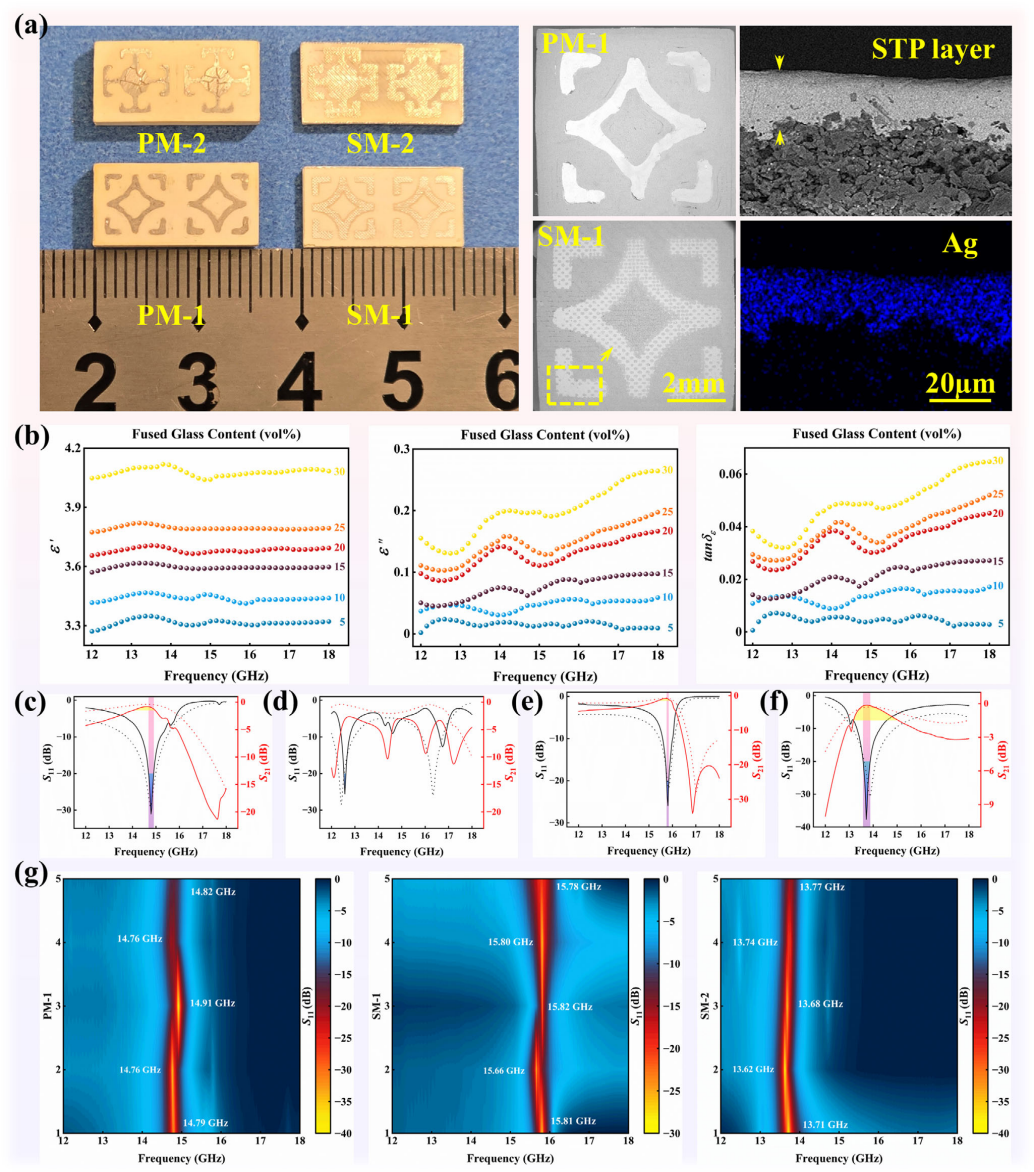
(a) Structures of BMFs; (b) *ε′*, *ε″*, and *tanδ_ε_* of substrates with different fused glass content; (c–f) Wave‐guide characterization results: (c) PM‐1; (d) PM‐2; (e) SM‐1; (f) SM‐2; (g) Measured *S*_11_ responses of five independently fabricated samples for each BMF configuration under identical experimental conditions.

Figure 4a presents the structures of four BMFs. A comparison of the two SRR fabrication routes reveals several process‐dependent limitations that are critical for scalability and reproducibility. In the silver‐filling approach, structural integrity depends strongly on SRR geometry. Narrow‐gap features (PM‐1) can be reliably filled, whereas larger continuous metal regions or wider apertures (PM‐2) exhibit penetrating cracks in the central disk. These defects originate from excessive silver paste accumulation and sintering‐induced stress concentration and were consistently observed across samples, defining a fabrication tolerance window and potentially limiting reproducibility for more complex designs. Interfacial and microstructural imperfections, such as two‐phase separation at SRR edges, further increase IL due to scattering and localized resistance. Controlling paste viscosity, filling strategy, and sintering profile is therefore critical for consistent performance. In contrast, the STP‐based process provides improved geometric definition and uniformity. CLR patterns fabricated by STP show slightly increased linewidth *W* due to screen‐printing tolerances, which may shift *f*_0_ toward lower frequencies. Residual mesh patterns indicate that transfer precision depends on screen mesh density. Cross‐sectional analysis confirms uniform Ag distribution with ∼20 µm penetration, demonstrating stable metal‐ceramic integration. Overall, silver filling allows complex SRR geometries with flexible linewidth control but is sensitive to stress and cracking, whereas STP supports ultra‐thin substrates, scalable fabrication, and reproducible SRR structures, albeit with moderate linewidth deviations. These two routes therefore provide complementary advantages in structural complexity, dimensional tolerance, manufacturability, and reproducible fabrication of different SRR configurations.

As shown in Figure 4b, introducing different percentages of fused glass as a sintering aid into the SiO_2_ substrate within Ku‐band causes *ε* *′*, *ε* *″*, and *tanδ_ε_* to increase in response to the content. At the macro level, the higher *ε′* and *ε″* of the molten fused glass are superimposed onto the SiO_2_ background. At the microscopic level, the main polarization mechanisms of the substrate should be attributed to the polarization of electrons and partial ion polarization with relatively fast response speeds. When the substrate is affected by an alternating *E*‐field in Ku‐band, the electron cloud of the atoms inside it will slightly shift relative to the atomic nucleus. This microscopic dipole moment caused by the deformation of the electron cloud lags behind in response to changes in the *E*‐field. Complete ion polarization in the glass phase is difficult, but the lattice oscillation of local ions can still contribute considerable ion polarization. The above factors jointly lead to the increase in the glass phase content response of the substrate's *ε′*, *ε″*, and *tanδ_ε_*. Additionally, this improvement is nonlinear. This is because at a low glass phase content (5–15 vol%), there are fewer composite interfaces within the substrate, and the increase in dielectric loss caused by electron and ion polarization is limited, with a slightly more significant increase in the real part. With the further increase in the introduction amount (15–25 vol%), while the interface increases, the synchronous content of non‐bridging O^−^ introduced by the glass phase also increases. Meanwhile, *ε′* and *ε″* increase significantly under the dual‐polarization effect. When the glass phase is sufficient to form a continuous interface within the substrate (>25 vol%), the local *E*‐field is uneven, and the loss caused by ionic lattice oscillation is obvious, ultimately manifested as a significant increase in *ε*.

Figure 4c–f presents the *S*‐parameters of four BMFs in Ku‐band. The glass phase additive, primarily consisting of B_2_O_3_ and Al_2_O_3_, acts as a sintering aid for SiO_2_. Due to lattice defects in the fused glass, these defects create conductive paths for leakage currents, which accumulate at the interface of the multiphase. This accumulation leads to interface polarization relaxation, contributing to increased substrate loss [57, 58]. The passband characteristics and *f*_0_ of the BMFs are primarily influenced by the constituent materials and UC dimension [59, 60]. Therefore, variations in the equivalent *LC* circuits result in distinct filtering functions. For PM‐1 (Figure 4c), the measured *f*_0_ exhibits a minimal deviation of 0.02 GHz (≈ 0.14% relative to simulated), with a passband spanning 14.7–14.9 GHz. Although the first resonant peak of *S*_11_ agrees well with the simulation, PM‐2 (Figure 4d) failed to maintain effective transmission due to penetrating cracks observed in the SRR structure (Figure 4a) and was therefore excluded from the quantitative *f*_0_ comparison. In contrast, the STP‐fabricated structures exhibit improved performance. For SM‐1 (Figure 4e), the measured passband ranges from 15.76 to 15.85 GHz (relative bandwidth 1.5%), with *f*_0_ at 15.81 GHz showing a deviation of 0.04 GHz (≈ 0.25%), *S*_11_ = −25.93 dB, and *S*_21_ = −0.89 dB. For SM‐2 (Figure 4f), the passband spans 13.59–13.88 GHz (relative bandwidth 4.8%), with *f*_0_ at 13.71 GHz deviating by 0.16 GHz (≈ 1.15%), *S*_11_ = −37.65 dB, and *S*_21_ = −0.13 dB. Overall, the frequency deviations of the functional BMFs remain within ∼1.2%, demonstrating good agreement between simulation and experiment and confirming the dimensional accuracy and reliability of the proposed fabrication processes.

Five independently fabricated samples were prepared for each BMF configuration and measured under identical conditions. The heat maps in Figure 4g show consistent resonance behavior across samples, with minor variations in *f*_0_ and *S*_11_. These results confirm the reproducibility of the fabrication process and the stability of the metal‐ceramic interface. Observed frequency deviations mainly originate from minor fabrication tolerances during printing and metallization, rather than interface instability, indicating reliable interfacial adhesion between the printed ceramic substrate and the SRR layer.

### 2.3 Filtering Mechanism

**FIGURE 5:**
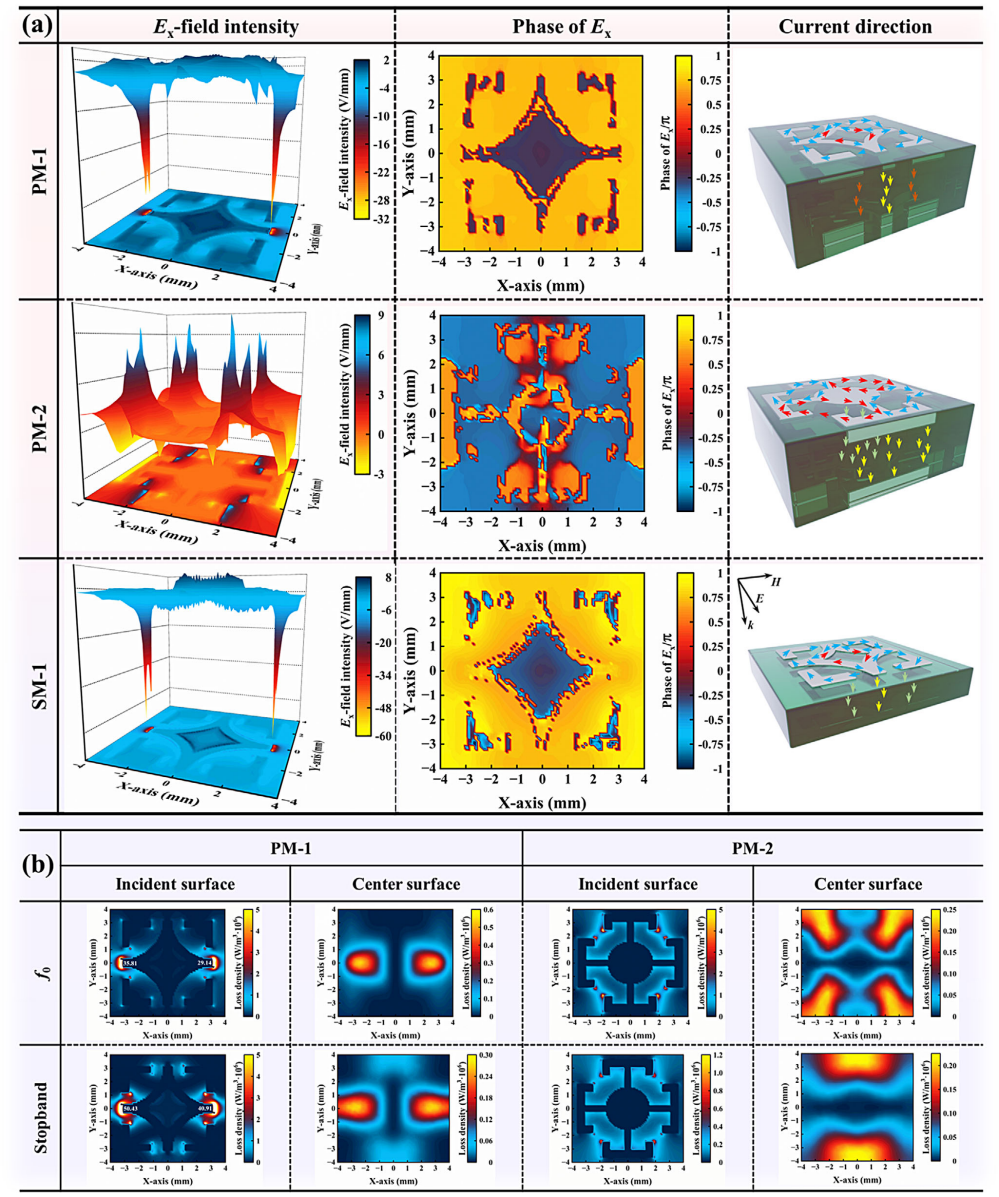
(a) Simulated *E*_x_‐field intensity distribution (V/mm) and phase of *E*_x_ (phase/π) at *f*_0_ for different BMF configurations, together with the corresponding current directions. The phase is shown relative to the incident plane wave and normalized to π (range: −1 to 1). (b) Energy loss density distributions (W/m^3^) at *f*_0_ and in the stopband for PM‐1 and PM‐2.

Figure 5a shows the simulated *E_x_*‐field intensity and phase distribution for the PM‐1, PM‐2, and SM‐1 at *f*_0_. Owing to the mirror symmetry of the UC, only the incident surface is discussed. The *E*‐field intensity reflects the strength of induced currents within the resonator, while the phase distribution provides insight into the dynamic interaction between inductive and capacitive components. Together, these results clarify the dominant filtering mechanisms of different BMF configurations. For PM‐1, the CLR generates a clockwise circulating current concentrated in the closed loop and corner inductive elements, enhancing the local *E*‐field through capacitive coupling. This capacitance‐dominated mechanism promotes efficient transmission at *f*_0_. In contrast, the filtering behavior of PM‐2 relies on *LC* impedance matching, where currents in spiral arms and corner elements interact with the central disc to balance inductive and capacitive impedances, minimizing reflection and stabilizing current flow. For SM‐1, the reduced synchronization between substrate thickness and CLR geometry weakens the capacitive gain effect. Consequently, the weakened capacitive gain leads to stronger field localization and enhanced out‐of‐band suppression, thereby improving passband selectivity [61, 62].

Energy loss density, defined as the energy dissipation per unit volume through dielectric conductivity and polarization mechanisms, is a key indicator of the BMF's filtering performance (Figure 5b) [63, 64, 65]. At *f*_0_, PM‐1 exhibits pronounced energy dissipation in CLR capacitive regions, consistent with its capacitance‐dominated mechanism. In the stopband, increased capacitive impedance restricts current migration, shifting energy concentration toward the incident plane. For PM‐2, the impedance‐matched condition at resonance enables balanced energy distribution across inductive and capacitive regions, resulting in characteristic energy loss patterns distinct from PM‐1. Outside the passband, impedance mismatch suppresses induced currents differently in each configuration, confirming that structural variation governs resonance pathways and energy redistribution. Overall, CLR‐based designs operate through capacitance‐enhanced local field superposition, whereas CSR‐based designs rely on *LC* impedance matching, explaining differences in transmission efficiency and passband tunability. These insights support the design of Ku‐band filters for enhanced selectivity or broadband tunability, including radome‐integrated filters and lightweight aerospace communication systems.

### 2.4 Passband Adjustability Verification

**FIGURE 6:**
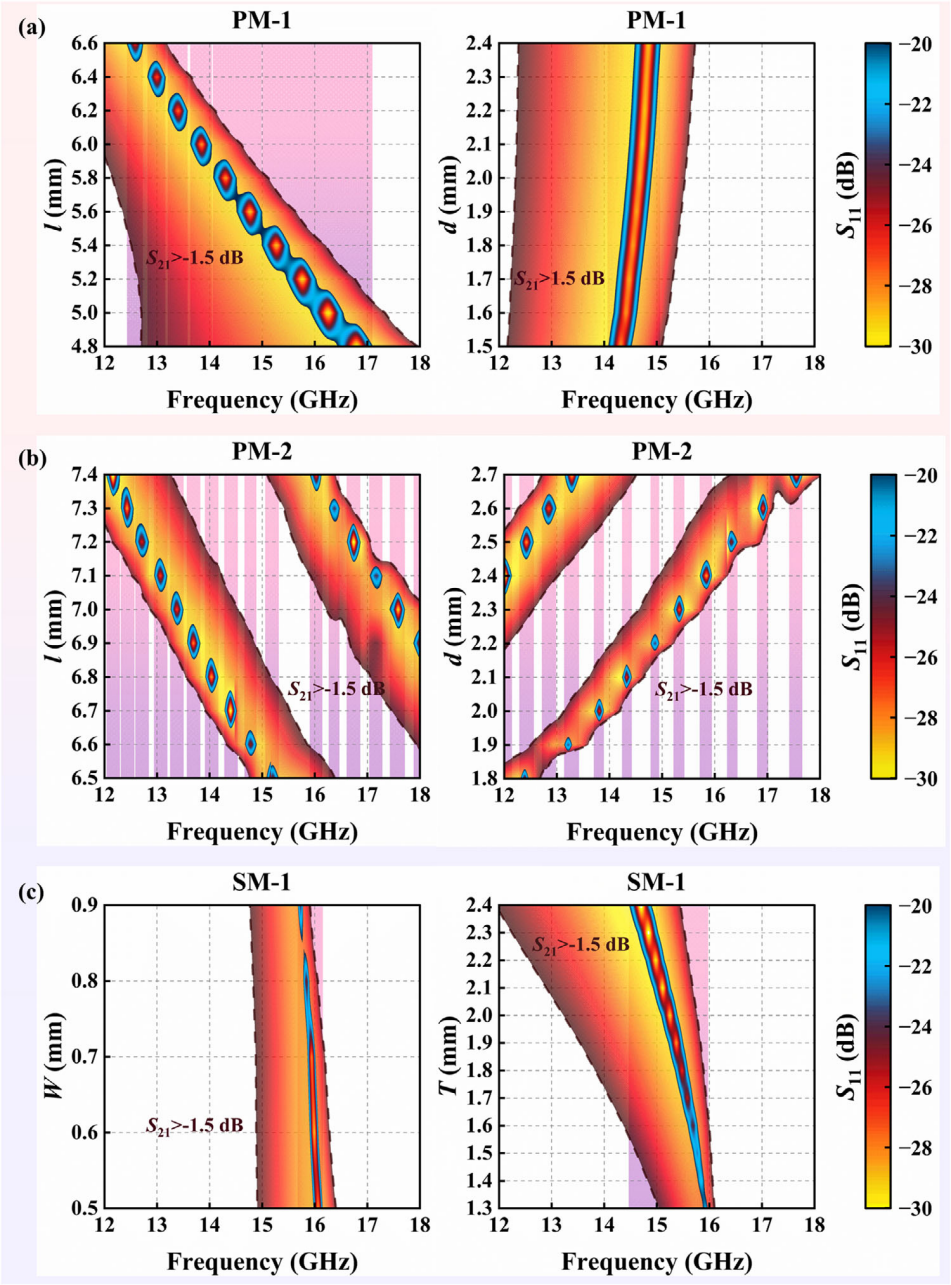
Passband adjustability verification: (a) Parameters *l* and *d* of PM‐1; (b) Parameters *l* and *d* of PM‐2; (c) Parameters *W* and *T* of SM‐1.

To demonstrate the tunability of the BMF passband and analyze the impact caused by processing errors, parametric studies were carried out for PM‐1, PM‐2, and SM‐1. The observed shift in *f*_0_, shown in Figure 6a–c, aligns with the relationship described in Equation (3), confirming the validity of the design models. Figure 6a illustrates the simulated tunable passband for PM‐1, which spans approximately 70% of the Ku‐band. This broad coverage demonstrates the high flexibility of the filter design. However, Figure 6a highlights that, due to the unique filtering mechanism of PM‐1, variations in the aperture width *d* exert a relatively minor effect on its *f*_0_, with an offset confined within 0.6 GHz. This observation underscores the capacitor‐dominated filtering mechanism in the CLR structure, where *d* primarily affects the capacitive characteristics but has limited influence on the overall frequency shift.

In contrast, the filtering behavior of PM‐2, which integrates both inductive and capacitive elements in its CLR design, shows a stronger dependence on the tuning of both *d* and *l*. Figure 6b shows that the PM‐2 achieves a dual‐passband structure within Ku‐band, covering approximately 65% of the band. This dual‐passband characteristic is a result of the combined inductive and capacitive filtering, allowing for multiple resonant frequencies. Tuning *d* in PM‐2 significantly influences *C* of the *LC* resonant circuit, resulting in a pronounced shift in the *f*_0_ offset. Owing to the leveling property of the silver paste, the *W* of CSR may increase unexpectedly. For SM‐1, as shown in Figure 6c, the increase of *W* causes *f*_0_ of SM‐1 to shift towards the low‐frequency direction, and this result is basically consistent with the functional relationship of Equation (3). Additionally, a passband structure within Ku‐band is observed when the substrate thickness *T* = 1.3 mm, aligning closely with the design objectives. With further increases in *T*, the passband width of SM‐1 expands, and *f*_0_ shifts toward X‐band. The practical fabrication constraints, especially during the STP process, resulted in a final thickness *T* of 1.4 mm.

The variations in *S*_11_ and *S*_21_ arise from multiple factors. *S*_11_ is highly sensitive to the *L* and *C* of the SRR, which influence both *f*_0_ and the reflection phase. During measurements, additional UC edge‐field coupling may be stronger than that in the simulation, which explains the *S*_11_ gain of BMFs. On the other hand, *S*_21_ is affected by substrate properties, interface conditions, and the testing environment. First, pyrolysis carbon from polymer decomposition increases substrate dielectric losses, inducing additional electromagnetic energy dissipation. Second, surface roughness and deviations in silver skin depth relative to ideal simulation lead to higher resistance at high frequencies, contributing to extra dielectric losses. Notably, PM‐2 shows more obvious *S*_21_ variations due to the cracked SRR structure. Third, minor detachment at the substrate‐metal interface, caused by incomplete filling of the silver paste or silver shrinkage during the filling process, introduces additional scattering losses, which were not accounted for in the simulations and contribute to higher measured IL. Furthermore, enhanced edge‐field coupling between adjacent UCs in the BMFs increases energy leakage, altering the equivalent impedance distribution and further contributing to *S*_11_ gain and *S*_21_ loss. Additional factors, such as connector contact resistance and vector network analyzer (VNA) cable losses, were also neglected in the simulations, which may further influence the measured *S*_11_ attenuation. These results illustrate the effectiveness of the designed unit cells in tuning the passband characteristics and demonstrate the potential for achieving efficient filtering while maintaining lightweight properties for practical applications.

### 2.5 Comparison of Existing Works

Table 1 summarizes a comparison of the proposed BMF structures with representative Ku‐band metamaterial bandpass filters reported in the literature, including key parameters such as IL, UC thickness (*λ*_L_), −3 dB passband, substrate type, and fabrication method. Most previously reported filters are implemented on planar microwave substrates such as FR‐4, Rogers laminates, or F4BM, and fabricated using standard PCB etching techniques. While these designs achieve reasonable IL and bandwidth, their electrical thickness (typically 0.051–0.14 *λ*_L_) is relatively large, constrained by the substrate and fabrication process. In addition, some reported designs are limited to simulations, restricting experimental validation and practical applicability.

**TABLE 1:** Comparison of Existing Works.

Ref.	IL (dB)	Thickness (*λ*_L_)	−3 dB Passband (GHz)	Substrate	Fabrication method
[24]	0.9	0.051	12.75–16.8	FR‐4	PCB
[66]	0.8	0.053	13.78–17.17	FR‐4	Simulation only
[67]	0.3	0.1	12–18	Rogers 5880LZ	Free‐space focused‐beam
[68]	0.34	0.058	17.25–21.59	F4BM	PCB
[69]	—	0.017	11.075–22.075	FR4	PCB
[70]	<0.15	0.072	24.72–30.24	Rogers RT5880	PCB
[71]	—	0.1	About 7–11	—	Simulation only
[72]	0.5	0.14	7.6–9.3	Rogers RO3003	Dielectric inserted plate
**This work (SM‐2)**	**0.13**	**0.06**	**12.75–16.86**	**SiO_2_**	**PEP‐STP**

In contrast, the present work employs PEP‐fabricated SiO_2_ ceramic substrates combined with STP metallization, reducing electrical thickness to 0.06 *λ*_L_. This thickness reduction modifies interlayer coupling and resonance behavior, enabling high transmission efficiency despite the reduced profile. As a result, the proposed BMF achieves a low IL of ∼0.13 dB with a broad operating band of 12.75–16.86 GHz within the Ku‐band. Ceramic additive manufacturing further enhances mechanical robustness and thermal stability compared to conventional polymer‐based substrates, making the design suitable for demanding environments.

## 3 Conclusions

This study successfully designed two novel SRR structures based on CLR and CSR, and developed four UC models using two SRR fabrication processes, including silver filling and STP. The BMF substrates were processed using 3D printing, and SRRs were integrated via these two fabrication techniques. Experimental results demonstrate that the silver filling process is effective for manufacturing SRRs with narrow linewidths, with the PM‐1 model exhibiting only a 0.33% deviation in *f*_0_ from the theoretical predictions. In contrast, the STP process offers higher structural precision and superior performance. The SM‐2 model, created with a UC thickness of 0.06 *λ*_L_, achieves an impressive transmission efficiency exceeding 97.05% and reflectance below 0.017% at the *f*_0_, closely matching the simulated *S*_21_ curve. The use of SiO_2_ substrates in combination with silver SRRs enhances the robustness of these metamaterials, making them suitable for extreme environmental conditions. Further simulations examining *E*‐field intensity, phase distribution, and energy loss mechanisms provide valuable insights into the bandpass filtering mechanism of the BMFs. The CLR structure relies on capacitance‐dominated transmission gain due to local field superposition, while the CSR structure achieves low‐loss electromagnetic transmission through inductive‐capacitive impedance matching. By carefully adjusting the geometric parameters of the UC, the filtering characteristics can be effectively tuned, achieving a passband tunability of up to 70% within Ku‐band. The proposed BMF platform demonstrates strong potential for radome‐integrated Ku‐band filters and lightweight aerospace communication systems. Its ultra‐thin ceramic architecture, high transmission efficiency, thermal stability, and high electromagnetic selectivity make it well‐suited for airborne and spaceborne platforms, establishing a scalable metamaterial filtering strategy for advanced electromagnetic communication applications.

## 4 Experimental Section

### 4.1 Simulation Method

A periodic structure model for the BMF unit cell (UC) was developed, focusing on the 12–18 GHz frequency range. According to the RE102 standard, the optimization criteria for the BMF included an IL below 1.5 dB and a reflection loss below −20 dB. The material parameters used in the simulation were experimentally determined: the dielectric constant of the substrate (*ε′* = 2.61, *ε″* = 0.015) was measured by the waveguide method. *S*‐parameters calculations were conducted using the frequency domain solver with open (add space) boundaries in the Z‐direction and UCs in the X‐ and Y‐directions. Subsequently, the distribution of the *E*‐field intensity and phase, as well as the energy loss density, which based on the simulated *S*‐parameters, were used to set the field monitor, with a data step size of 0.05 mm.

### 4.2 Preparation Process of BMFs

The IS PRM was prepared by combining 73.35 vol% ball‐milled inorganic powder (SiO_2_: fused glass = 9:1) with 26.65 vol% polymer binder and additives. This mixture was processed in a twin‐screw mixer (Kejing, China) at 180°C for 1.5 h. After cooling, the resulting material was crushed into granules averaging 2–3 mm in size. Following material preparation, four BMF STL models were imported into Uprise3D 3.48.108, converted into G‐code, and printed using the PEP printer (UPS‐250DA, Uprise3D, China). Printing parameters included a 0.1 mm layer height, 0.2 mm line width, 0.5 mm retraction distance, and 0.3 mm·s^−1^ retraction speed. Deposition was performed using a 0.2 mm nozzle at 165°C, employing an orthogonal infill strategy with alternating lines oriented at [−45°,45°]. Post‐processing consisted of degreasing, sintering, and SRR introduction. The PGBs were first subjected to solvent extraction in n‐hexane for 48 h to remove oils and waxes. The defatted PGBs were then heated in an air atmosphere at 500°C for 2 h to decompose the polymer skeleton. Sintering was followed at 800°C for 2 h to consolidate the substrate. Then, the silver paste was coated (Shanghai, China) on the surface of sintered PGB‐PM, and STP was performed on the surface of sintered PGB‐SM using transfer screens that matched the size of the two SRR 2D models. Finally, the silver paste diluent was dried at 120°C for 15 min, and then sintered at 720°C for 15 min to complete the preparation of BMFs.

### 4.3 Characterization

XRD (Bruker D8 Advance, Germany) was employed to characterize the phase composition of IS PRM, sintered PGB, and BMF samples. Elemental valence states were analyzed via XPS (Thermo ESCALAB 250X, USA). The microstructure and elemental distribution of the SRR plane were examined using a scanning electron microscope combined with energy‐dispersive spectroscopy (SEM, EDS mapping; Bruker, Germany). *S*‐parameters of four distinct BMFs were measured using a VNA (Agilent N5244A, USA) with a Ku‐band waveguide fixture. For testing, BMF samples were fabricated into standard Ku‐band rectangular waveguide specimens with dimensions of 15.9 mm × 7.9 mm × 3.0 mm.

## Funding

This work was financially supported by the Leading Talent Project of the National Special Support Program (No. 2022WRLJ003), National Natural Science Foundation of China (No. 52202126), and Shenzhen Science and Technology Program (Nos. 202206193000001, 20220818183014003).

## Conflicts of Interest

The authors declare no conflicts of interest.

## Data Availability

The data that support the findings of this study are available from the corresponding author upon reasonable request.
